# Health Impacts of Future Prescribed Fire Smoke: Considerations From an Exposure Scenario in California

**DOI:** 10.1029/2023EF003778

**Published:** 2024-02-07

**Authors:** Andrew Rosenberg, Sumi Hoshiko, Joseph R. Buckman, Kirstin R. Yeomans, Thomas Hayashi, Samantha J. Kramer, ShihMing Huang, Nancy H. F. French, Ana G. Rappold

**Affiliations:** 1 Environmental Health Investigations Branch, California Department of Public Health, Richmond, CA, USA; 2 California Epidemiologic Investigation Service Fellowship Program (Cal-EIS), Chronic Disease Control Branch, Center for Chronic Disease Prevention and Health Promotion, California Department of Public Health, Sacramento, CA, USA; 3 Sequoia Foundation, La Jolla, CA, USA; 4 Sonoma Technology, Inc., Petaluma, CA, USA; 5 Michigan Tech Research Institute, Michigan Technological University, Ann Arbor, MI, USA; 6 Center for Public Health and Environmental Assessment Office of Research and Development, U.S. Environmental Protection Agency, Research Triangle Park, NC, USA

## Abstract

In response to increasing wildfire risks, California plans to expand the use of prescribed fire. We characterized the anticipated change in health impacts from exposure to smoke under a future fire-management scenario relative to a historical period (2008–2016). Using dispersion models, we estimated daily fine particulate matter (PM_2.5_) emissions from hypothetical future prescribed fires on 500,000-acres classified as high priority. To evaluate health impacts, we calculated excess daily cardiorespiratory emergency department visit rates attributed to all-source PM_2.5_, distinguishing the portion of the burden attributed to prescribed fire. The total burden was differentiated by fire type and by smoke strata-specific days to calculate strata-specific burden rates, which were then applied to estimate the burden in the future scenario. This analysis suggests that the exposure to prescribed fire smoke, measured as the number of persons exposed per year, would be 15 times greater in the future. However, these exposures were associated with lower concentrations compared to the historical period. The increased number of exposure days led to an overall increase in the future health burden. Specifically, the northern, central, and southern regions experienced the largest burden increase. This study introduces an approach that integrates spatiotemporal exposure differences, baseline morbidity, and population size to assess the impacts of prescribed fire under a future scenario. The findings highlight the need to consider both the level and frequency of exposure to guide strategies to safeguard public health as well as aid forest management agencies in making informed decisions to protect communities while mitigating wildfire risks.

## Plain Language Summary

Prescribed fire is a forest management strategy for reducing the risks of wildfires. While some fires are ecologically beneficial, smoke from fires is a major source of airborne particle pollution, which is harmful to human health. This study examined the change in health impacts resulting from an expected increase in the use of prescribed fire within California’s high-priority wildfire risk areas. We used daily counts of cardiorespiratory emergency department visits attributed to air quality combined with model-generated measures of smoke pollution to estimate health impacts. We compared exposures and the associated health burden on days impacted by wildfire or prescribed fire smoke in the past to the impacts in the hypothetical future scenario with increased prescribed fire. Projections of future prescribed burning in high priority areas suggest that more people would experience smoke more often, although exposures would occur at lower concentrations. With more frequent lower-level exposure days near populated areas, the health burden would increase relative to past prescribed fire. Understanding the potential impact of prescribed fire may simultaneously help protect public health and increase safety from wildfires.

## 1. Introduction

Wildfire smoke is a significant contributor to airborne particle pollution (Jaffe et al., 2008, 2020; Liu et al., 2016; Liu & Peng, 2019; McClure & Jaffe, 2018) and a known health hazard (Black et al., 2017; Burke et al., 2021; Cascio, 2018; Reid et al., 2016; Reid & Maestas, 2019). In recent years, wildfire emissions have had a dramatic impact on air quality and health (Abatzoglou & Williams, 2016; Burke et al., 2021; Jaffe et al., 2020). Extreme weather, such as high seasonal temperatures and extended droughts, have intensified the frequency, severity, and duration of wildfire events particularly in the Western US (Spracklen et al., 2009; Westerling, 2016; Westerling & Bryant, 2007; Williams et al., 2019). In recognition of the climate-driven wildfire crisis and the need to restore healthy forests in California, state and federal agencies entered into a historic shared stewardship agreement to treat 1 million acres per year of forest and wildlands annually by 2025 (State of California & USDA Forest Service Pacific Southwest Region, 2020).

Prescribed fires (i.e., prescribed burning) are landscape fires intentionally set under specific conditions to reduce the intensity and spread of uncontrolled fire, and to provide other benefits such as improved watershed management and forest ecosystem health (Hunter & Robles, 2020; U.S. EPA, 2020a). The stewardship agreement established a framework for forest management agencies to greatly expand the use of prescribed fire across California’s landscape. Current goals aim to treat 400,000 to 500,000 acres of lands with beneficial fire per year, a significant increase from previous years’ estimates of approximately 100,000 acres of prescribed fire per year, which have been rising more recently (California Wildfire & Forest Resilience Task Force, 2022; Kelp et al., 2023; LAO, 2022). Prescribed and cultural burns are also conducted by local entities, such as tribal governments and private landowners (California Wildfire & Forest Resilience Task Force, 2022). Therefore, characterizing the air quality and public health impacts posed by increasing the frequency and scale of prescribed burning may provide guidance on the implementation of its expanded use.

Increasingly, wildfire smoke emissions have countered trends in air quality improvements and related health benefits, particularly in western states (McClure & Jaffe, 2018). Burke et al. (2021) estimated that in western regions up to 50% of the ambient mass concentration of particulate matter (PM) is fire-originated and mixed with emissions from other sources. Fine particulate matter (PM_2.5_) is a known risk factor for cardiorespiratory morbidity and all-cause mortality (Cohen et al., 2017; EPA, 2020b; Pope et al., 2020) and has been associated with a number of other outcomes (Cleland et al., 2022; Ebisu & Bell, 2012; Hansen et al., 2016; Jerrett et al., 2013; Kloog et al., 2012). Health risks associated with acute exposure to wildfire smoke-attributed PM_2.5_ have been widely reported both globally and within the US (Alman et al., 2016; Cascio, 2018; Chen et al., 2021; DeFlorio-Barker et al., 2019; Delfino et al., 2009; Dennekamp et al., 2015; Gan et al., 2017; Haikerwal et al., 2015; Johnston et al., 2007; Jones et al., 2020; Liu et al., 2017; Rappold et al., 2012; Wettstein et al., 2018).

As the use of prescribed burning increases, understanding how smoke emissions and the corresponding health impacts compare to past levels can inform future mitigation efforts, fire and land management decisions, and public health planning. Recent studies have begun to characterize differential PM_2.5_ impacts between wildfire and prescribed fire in the Western US (EPA, 2021; Kelp et al., 2023). However, few epidemiological studies have investigated health effects specifically attributed to prescribed fire smoke (Afrin & Garcia-Menendez, 2021; EPA, 2021; Huang et al., 2019; Prunicki et al., 2019). Two prescribed fire health studies were conducted in Georgia (Afrin & Garcia-Menendez, 2021; Huang et al., 2019), although southeastern climate and topography differ greatly compared to the Western US, as do their prescribed fire regimes. Another recent assessment conducted by the US Environmental Protection Agency (EPA) compared estimated health impacts attributed to historical and hypothetical wildfire and prescribed fire scenarios in two case studies using health impact functions from previous wildfire epidemiological studies (EPA, 2021). To our knowledge, no other study has investigated future anticipated health impacts associated with increased prescribed burning.

This study compared the air quality impacts of PM_2.5_ emissions from wildfire and prescribed fire smoke on the California population in recent years to a hypothetical future prescribed fire scenario. In this scenario, prescribed burning emissions were simulated to reflect the statewide target of increasing prescribed fire to 500,000 acres per year, covering 4 million acres in total (Kramer et al., 2023). The study developed health impact functions for cardiorespiratory emergency department visits statewide and characterized the excess health burden attributed to prescribed fire smoke. To evaluate the projected change in the prescribed fire smoke-related cardiorespiratory burden, the smoke-attributed burden from prescribed fires in the historical period was compared to the burden in the future prescribed fire scenario.

## 2. Data and Methods

### 2.1. Historical Wildfire PM_2.5_ and Prescribed Fire PM_2.5_ Data

Daily average wildfire- and prescribed fire-derived PM_2.5_ concentrations were modeled using the United States Forest Service BlueSky smoke modeling framework for emission characteristics (fuel beds, fuel moisture, emissions, time profile, plume rise) with Hybrid Single-Particle Lagrangian Integrated Trajectory (HYSPLIT) dispersion modeling (Kramer et al., 2023; McClure et al., 2023). BlueSky was initialized with integrated satellite-fire detections and historical fire records. Meteorological data from the North American Mesoscale Forecast System Model (NAM) 12-km resolution forecasts were applied to simulate the dispersion of PM_2.5_ from wildfire and prescribed fire emissions.

For computational feasibility, dispersion was specified with a maximum particle retention of 5 days for wildfire emissions and 2 days for prescribed fire emissions. The default value of 5 days for wildfire implemented in BlueSky was sufficient to capture wildfire smoke transport within the California domain. Sensitivity analyses determined that two days was sufficient for prescribed fire as, after 48-hr, the remaining smoke was highly dispersed and low in concentration (Kramer et al., 2023). Model output provided daily average smoke PM_2.5_ at 12-km horizontal resolution for the 0–500-m atmospheric layer. Sensitivity testing showed that 500-m is a representative height for the top of the “surface layer” to represent daily average boundary height conditions.

Model predictions of smoke PM_2.5_ concentrations were downscaled to 1-km^2^ gridded resolution using bilinear interpolation, and daily ZIP code average estimates of wildfire PM_2.5_ and prescribed fire PM_2.5_ concentrations were calculated for each California ZIP code using zonal statistics. Hereafter wildfire- and prescribed fire-derived PM_2.5_ concentrations (μg/m^3^) are referred to as WF-PM_2.5_ and Rx-PM_2.5_. The HYSPLIT-estimated concentrations differ from a PM_2.5_ as they do not include emissions of PM_2.5_ from other ambient and background sources. Evaluation of HYSPLIT concentration estimates found reasonable correlations with monitored data both with and without NOAA’s Hazard Mapping System (HMS) as a filter (See SI for evaluation details; Kramer et al., 2023).

### 2.2. Projected Future Prescribed Fire PM_2.5_ Concentrations Under a Target Burn Scenario

Daily average prescribed fire PM_2.5_ concentrations were modeled for a hypothetical future fire management scenario that projects increased prescribed burning up to 500,000-acres per year across California (Kramer et al., 2023). Prescribed fire simulations were specifically applied to lands designated by the California Department of Forestry and Fire Protection (CAL FIRE) as high priority, based on high wildfire hazard risk and housing density (Community Wildfire Threat Priority Landscape Classes 4 and 5) (FRAP, 2018). To attain a comprehensive understanding of the implications of prescribed fire, we deliberately excluded lands burned for agricultural purposes from this analysis. By adopting these modeling choices, we present an aggressive fire management scenario that achieves the target and leads to more direct population exposures, resulting in a more conservative health-protective analysis.

Hypothetical prescribed burns were simulated using random ignition points within the CAL FIRE-designated high wildfire risk landscapes. The distribution of vegetation types of the hypothetical prescribed fires was driven by the vegetation types in these designated locations. Fire size was assigned to these hypothetical prescribed fires by vegetation type, following the fire size distribution of each vegetation type based on a historical fire inventory developed from satellite-derived data products and agency fire records. Burn days were randomly assigned to these hypothetical prescribed fires to allowable burn dates in 2014 using daily burn decision records obtained from the California Air Resources Board. The hypothetical prescribed fires covered 4 million acres total, distributed over eight annual cycles (years) to meet the 500,000-acres per year target. Details of the target scenario development may be found in Kramer et al. (2023).

Meteorological data from NAM was used to model the dispersion of PM_2.5_ from hypothetical prescribed fire emissions using BlueSky and HYSPLIT with the exact model parameters for particle retention and height as defined in the historical scenario. To achieve consistency in modeling across all target cycles, meteorological data for 2014 was selected as a representative fire weather year for carrying out prescribed fires because it had the median number of statewide prescribed fire burn days and the least variation in key meteorological parameters among the years 2008–2017 (NAM 12-km data). Monthly wind speed, relative humidity, precipitation, and soil moisture values for 2014 were less than two standard deviations of the 2008–2017 average of each parameter. Model predictions of PM_2.5_ concentrations were downscaled to 1-km^2^ gridded resolution using bilinear interpolation. Daily ZIP code average estimates of future projected prescribed fire PM_2.5_ concentrations were calculated for each California ZIP code using zonal statistics. Future projected prescribed fire-derived PM_2.5_ concentrations (μg/m^3^) are hereafter referred to fRx-PM_2.5_.

### 2.3. Identification of “Smoke Days” and “Smoke Strata”

We used HYSPLIT estimates of PM_2.5_ to characterize exposure on each day in each ZIP code as “smoke days” (i.e., impacted by wildfire, prescribed fire, or both) and “smoke strata.” The smoke concentration strata corresponded to no smoke (0), 0.01–0.05, 0.06–0.10, 0.11–0.25, 0.26–0.50, 0.51–1.00, 1.01–5.00, 5.01–10.00, 10.01–15.00, 15.01–20.00, 20.01–50.00, 50.01–100.00, 100.01–150.00, and 150.01+ μg/m^3^. In the historical period, the maximum wildfire concentration was 1120.00 μg/m^3^ and the maximum prescribed fire concentration was 253.00 μg/m^3^ (Summary statistics of exposure distributions are presented in Tables S1 and S2 in Supporting Information S1). In addition, we define population exposure as the number of persons exposed each day (person-days), capturing the cumulative number of persons exposed across all smoke days, by smoke strata, and fire type.

### 2.4. Study Population and Health Data

Statewide emergency department (ED) visit records were obtained from the California Department of Health Care Access and Information (HCAI) for the period between 1 January 2008, and 31 December 2016 (“historical period”). ED visits related to respiratory disease (asthma, chronic obstructive pulmonary disease, and other conditions) and cardiovascular disease (myocardial infarction, hypertension, and other conditions) were identified using the International Classification of Diseases ninth revision and tenth revision Clinical Modification (ICD-9-CM and ICD-10-CM) diagnosis codes. Details on the specific disease codes have been previously reported (Thilakaratne et al., 2022). Visit-level data were aggregated by date and ZIP code of residence to produce daily ZIP code-level counts of each health outcome. ZIP code population sizes were based on 2010 census data. ZIP codes with a population size less than 1,500 (1.9% of the California population) were excluded due to concerns regarding model performance.

### 2.5. Air Pollution Data (Ambient PM_2.5_)

Daily average ambient PM_2.5_ concentrations (μg/m^3^) were obtained from a previously developed high-resolution spatiotemporal ensemble model (Di et al., 2019). Briefly, the ensemble model integrated machine learning algorithms including neural networks, random forest, and gradient boosting, and a geographically weighted generalized additive model to predict daily ambient PM_2.5_. The ensemble-based model was calibrated using satellite-retrieved data, chemical transport model simulations, land-use variables, meteorological variables, and EPA Air Quality System measurements. This approach yielded strong model performance, with an average cross-validation R^2^ of 0.86 for daily predictions of PM_2.5_ (Di et al., 2019). Daily (24-hr mean) 1-km^2^ gridded PM_2.5_ estimates were interpolated at census tract-level and further population-weighted for each ZIP code to obtain daily averages of total ambient PM_2.5_ in California from 1 December 2007, to 31 December 2016. Ambient PM_2.5_ represents the total undifferentiated particle concentration derived from all sources, hereafter referred to as “aPM_2.5_.”

### 2.6. Estimating Risk Associated With aPM_2.5_ and Cardiorespiratory Health Outcomes

Details on the statistical models used to estimate associations between cardiorespiratory outcomes and aPM_2.5_ have been previously reported in Thilakaratne et al. (2022). Briefly, Poisson regressions were applied to estimate the relative risk associated with aPM_2.5_ and daily counts of ED visits for respiratory and cardiovascular outcomes separately. The models were adjusted for known time-varying confounders, including temperature, relative humidity, seasonality, day-of-the-week, and a random intercept was applied to each ZIP code. Distributed lag models were used to estimate acute effects cumulatively up to 4 days (lag 0–4) following each day of exposure for respiratory outcomes and on the same day of exposure for cardiovascular outcomes. The length of lag periods for each outcome model was selected based on the best model fit using the Bayesian Information Criterion. Using the best-fit model, we estimated that a 10 μg/m^3^ increase in aPM_2.5_ was associated with 2.27% (95% CI: 2.14, 2.39) and 0.89% (95% CI: 0.80, 0.98) in respiratory and cardiovascular-related ED visits, respectively (Thilakaratne et al., 2022).

Converted to daily average baseline rates, the observed number of ED visits during the study period was 11.5 per 100,000 persons for respiratory, and 12.6 per 100,000 (hereafter 100K) for cardiovascular-related visits per day. Applying the risk coefficients for respiratory and cardiovascular outcomes to the time-series of aPM_2.5_ concentrations and observed ED visits, we calculated the aPM_2.5_-attributable number of ED visits across all days and ZIP codes (ZIP-days). The daily average aPM_2.5_ burden rates, which reflected the ambient PM_2.5_-attributed rate of ED visits per 100,000 persons per day, were 0.25 per 100K and 0.11 per 100K for respiratory and cardiovascular-related outcomes, respectively. The overall baseline rate of respiratory morbidity was lower than cardiovascular morbidity; however, given the larger magnitude of the relative risk, the aPM_2.5_-attributed respiratory burden was larger than that of the aPM_2.5_-attributed cardiovascular burden.

The health impact functions were associated with total ambient PM_2.5_ concentrations (aPM_2.5_), encompassing the magnitude of exposure, measure of risk, and population size. The aPM_2.5_-attributable number of respiratory and cardiovascular outcomes were summed together and expressed as cardiorespiratory burden rates per 100,000 persons.

### 2.7. Estimation of PM_2.5_-Associated Health Burden for Ambient PM_2.5_, Wildfire, and Prescribed Fire Smoke

The aPM_2.5_-attributed health burden was compared by concentrations of modeled smoke emissions. The HYSPLIT models do not account for PM_2.5_ emissions from other sources of pollution, challenging the ability to validate against monitored values of total ambient concentrations. Therefore, to link the concentration-dependent aPM_2.5_ burden with HYSPLIT smoke strata, daily aPM_2.5_-attributed burden rates were stratified by fire type (i.e., whether impacted by wildfire, prescribed fire, or future prescribed fire smoke) and by smoke strata, including a zero-smoke stratum (Section 2.3). To obtain statewide “strata-specific aPM_2.5_ burden rates”, we first added the excess number of aPM_2.5_-attributed ED visits for each ZIP code and smoke stratum and divided by the corresponding number of days in each stratum (average incidence per smoke strata in a ZIP code). Second, we then divided the strata-specific daily incidence by the corresponding population size and multiplied by 100,000. Finally, we averaged daily rates within each smoke stratum (across all affected ZIP codes).

To estimate strata-specific daily aPM_2.5_ burden rates for the hypothetical future prescribed fire (fRx) scenario, we applied the aPM_2.5-_attributed ED visit burden rates from the historical period to the projected prescribed fire smoke ZIP-days, matched by day of the year. The aPM_2.5-_attributed burden for the future scenario was summed across days, and an average incidence rate was calculated by dividing by the number of projected smoke days in each ZIP code stratum and multiplying by the ZIP code population size. This resulted in a daily burden rate for each ZIP code and stratum. Lastly, these daily rates were averaged within each smoke stratum and multiplied by 100,000.

### 2.8. Annual Prescribed Fire Smoke Burden Rates

To assess the cumulative health impact related to prescribed fire annually, we compared the burden attributable to prescribed fire smoke in each scenario. For each ZIP-day impacted by prescribed fire smoke in the historical period, we estimated the aPM_2.5_-attributed ED visit burden, had there been no prescribed fire smoke, by applying the zero-smoke stratum daily burden rates. The burden specifically attributed to prescribed fire smoke was then estimated for each ZIP-day as the difference between the aPM_2.5-_attributed ED visit burden and the corresponding counterfactual, zero-smoke aPM_2.5_-attributed burden rate. The difference in burden was then summed across all smoke days within the ZIP code, divided by the population size, and multiplied by 100,000.

In the future scenario, the prescribed fire smoke-attributed burden was characterized as the difference between the estimated aPM_2.5-_attributed burden on projected prescribed fire smoke days and the counterfactual, zero-smoke burden rate on the corresponding ZIP-days. Finally, the change in annual burden rates between the historical and future scenarios was evaluated by calculating the difference in prescribed fire smoke-attributed burden between the historical period and future scenario for each ZIP code (difference in difference).

## 3. Results

### 3.1. Smoke-Derived PM_2.5_ and Total Ambient PM_2.5_ Exposure Concentrations and Smoke Days

**Figure 1.**
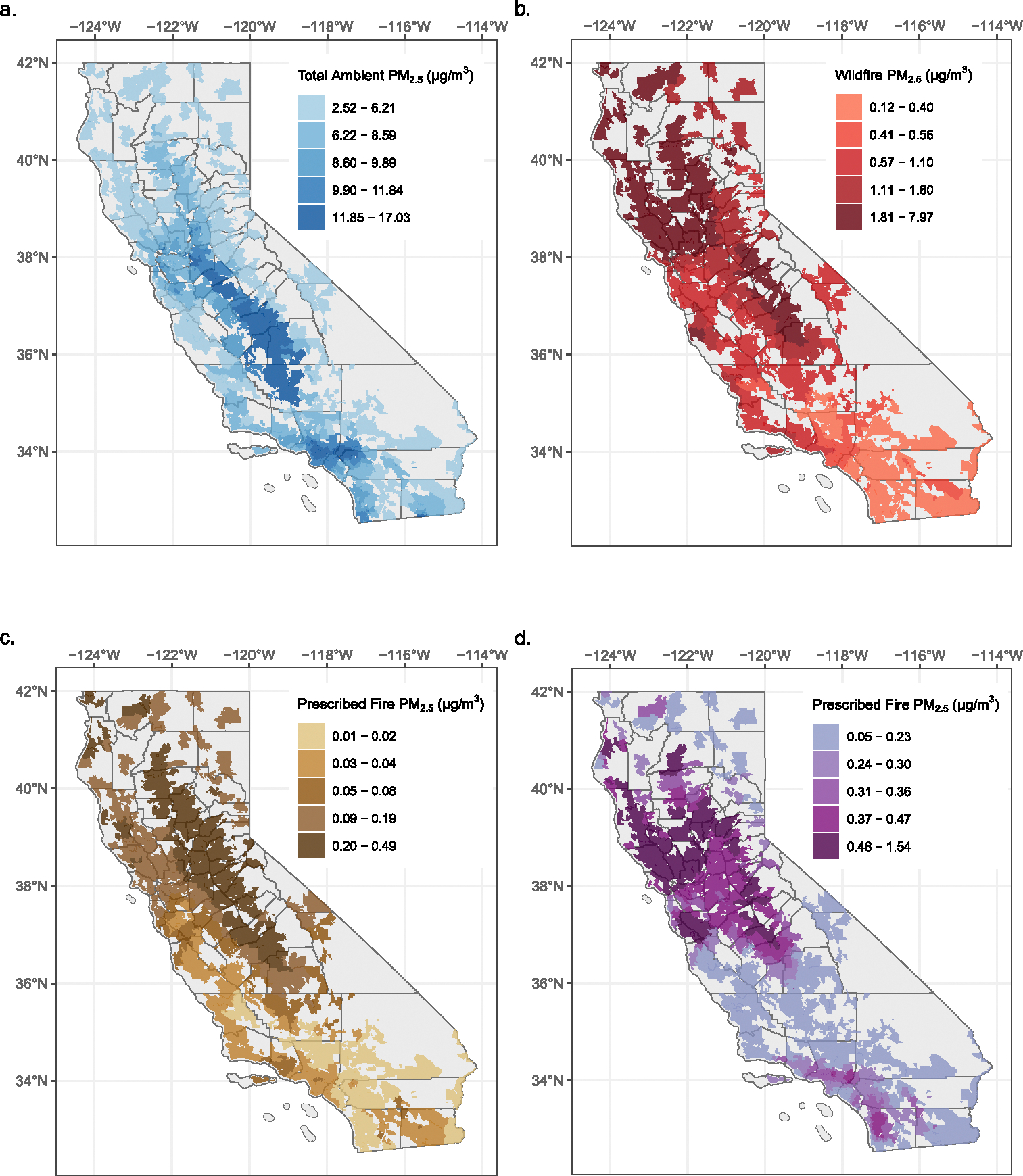
Maps of Fine Particulate Matter (PM_2.5_) Concentrations (μg/m^3^) during the Historical Period (2008–2016) and Future Prescribed Fire Scenario. Historical daily mean concentrations are shown for (a) Total ambient PM_2.5_ (aPM_2.5_), (b) Hybrid Single-Particle Lagrangian Integrated Trajectory (HYSPLIT)-modeled wildfire PM_2.5_ (WF-PM_2.5_) for the Historical Period, and (c) HYSPLIT-modeled prescribed fire PM_2.5_ (Rx-PM_2.5_) for the Historical Period. Future projected daily mean concentrations for HYSPLIT-simulated prescribed fire PM_2.5_ (fRx-PM_2.5_) are shown in (d). Breaks in colors correspond to the quintiles of respective exposure distributions.

From 2008 to 2016, daily ambient PM_2.5_ (aPM_2.5_) averaged 9.11 ± 6.90 (SD) μg/m^3^ across 1,297 ZIP codes, with the highest concentrations observed in the Central Valley and inland areas of Los Angeles (Figure 1a). During the same period, modeled WF-PM_2.5_ concentrations averaged 1.12 ± 9.21 μg/m^3^ and Rx-PM_2.5_ concentrations averaged 0.11 ± 1.26 μg/m^3^. Due to regional variability in smoke emissions and the intensity of emissions, exposure to smoke PM_2.5_ was not evenly distributed across the state. The northern and central regions of the state experienced the highest levels of WF-PM_2.5_ and Rx-PM_2.5_ exposure (Figures 1b and 1c). Geographically, the distribution of smoke days did not align with exposure impacts based on magnitude. Regions with the greatest number of days impacted by wildfire smoke included the central and southern region, whereas the central and northern regions experienced more days impacted by prescribed fire smoke.

Under the future prescribed fire scenario, modeled fRx-PM_2.5_ concentrations were similar in magnitude to historical levels, averaging 0.36 ± 0.95 μg/m^3^ per day (historical Rx-PM_2.5_: 0.11 ± 1.26 μg/m^3^) (Figure S1 in Supporting Information S1). The northern-central and northern-coastal regions had the highest estimated levels of exposure in the future scenario, whereas the central and southern-coastal regions experienced more smoke days (Figure 1d; Figure S2 in Supporting Information S1). Distributions of ambient PM_2.5_ and fire-specific PM_2.5_ concentrations, as well as the cumulative number of ZIP-days impacted by smoke for each concentration stratum, are provided in Supplemental Materials (Tables S1–S4 in Supporting Information S1).

We observed differences in exposure patterns due to geographical and seasonal variations in prescribed burning activity in the past. Compared to wildfires, higher intensity prescribed burns (>5.0 μg/m^3^) occurred, on average, in less population-dense areas (mean population density: 2,859 persons/sq. mile (prescribed fire) versus 4,108 persons/sq. mile (wildfire). Additionally, peak planned burn activity occurred between November and January, whereas peak wildfire activity occurred between June and September.

### 3.2. Population Impacts From Wildfire- and Prescribed Smoke-Related PM_2.5_

**Figure 2.**
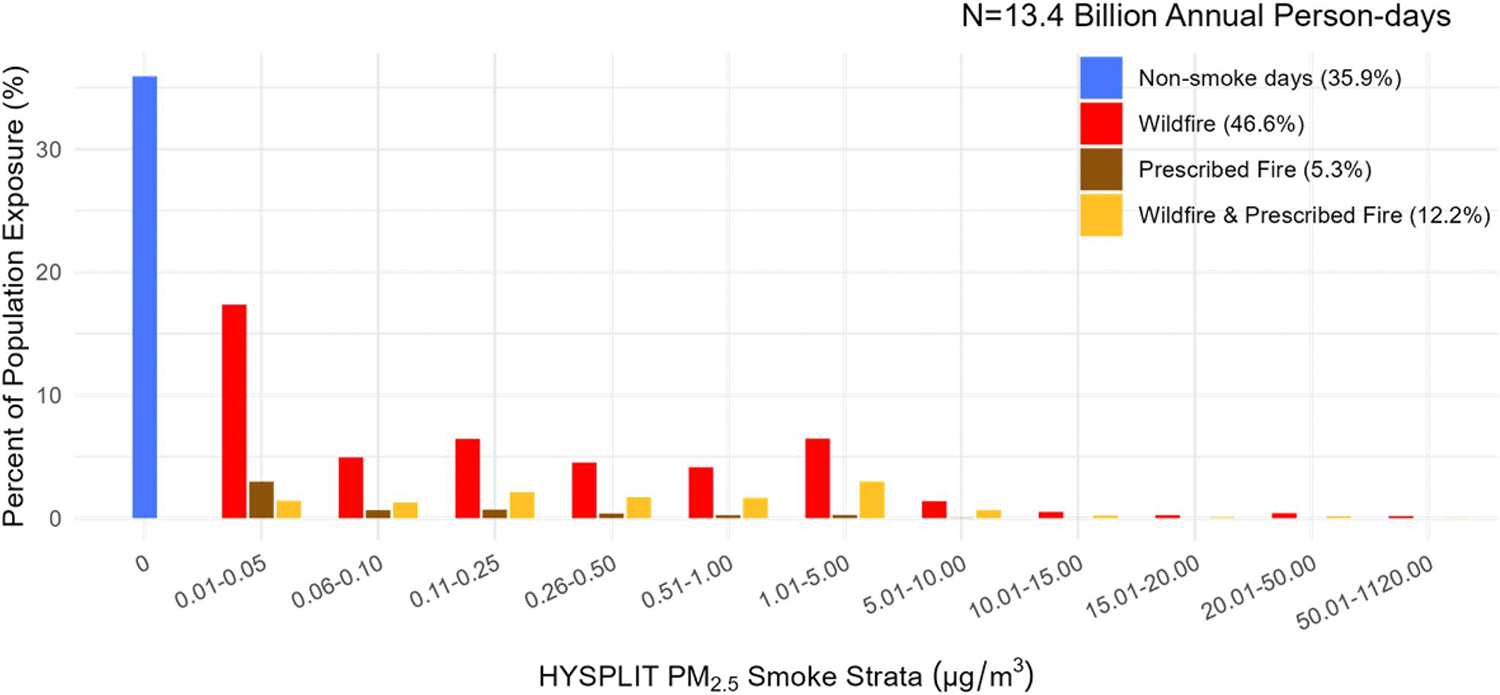
Fraction of Population Exposure (in Person-days) in the Historical Period (2008–2016). Population Exposure is measured in person-days and expressed as the total number of persons exposed between 2008 and 2016. The percent of total person-day exposure is broken down by Hybrid Single-Particle Lagrangian Integrated Trajectory-modeled PM_2.5_ Smoke Strata (μg/m^3^) and by fire type.

Among 13.4 billion annual person-days of exposure during the historical period, wildfire-impacted days accounted for the largest fraction of exposure (46.6%), whereas prescribed fires accounted for the least (5.3%) (Figure 2). On wildfire smoke-impacted days (WF smoke days), about 6% of the exposure occurred on days when wildfire smoke concentrations exceeded 5.0 μg/m^3^ (WF-PM_2.5_ >5.0). In contrast, less than 1.2% of person-days of prescribed fire (Rx-PM_2.5_) exposure occurred when concentrations were above this level (Table 1).

**Figure 3.**
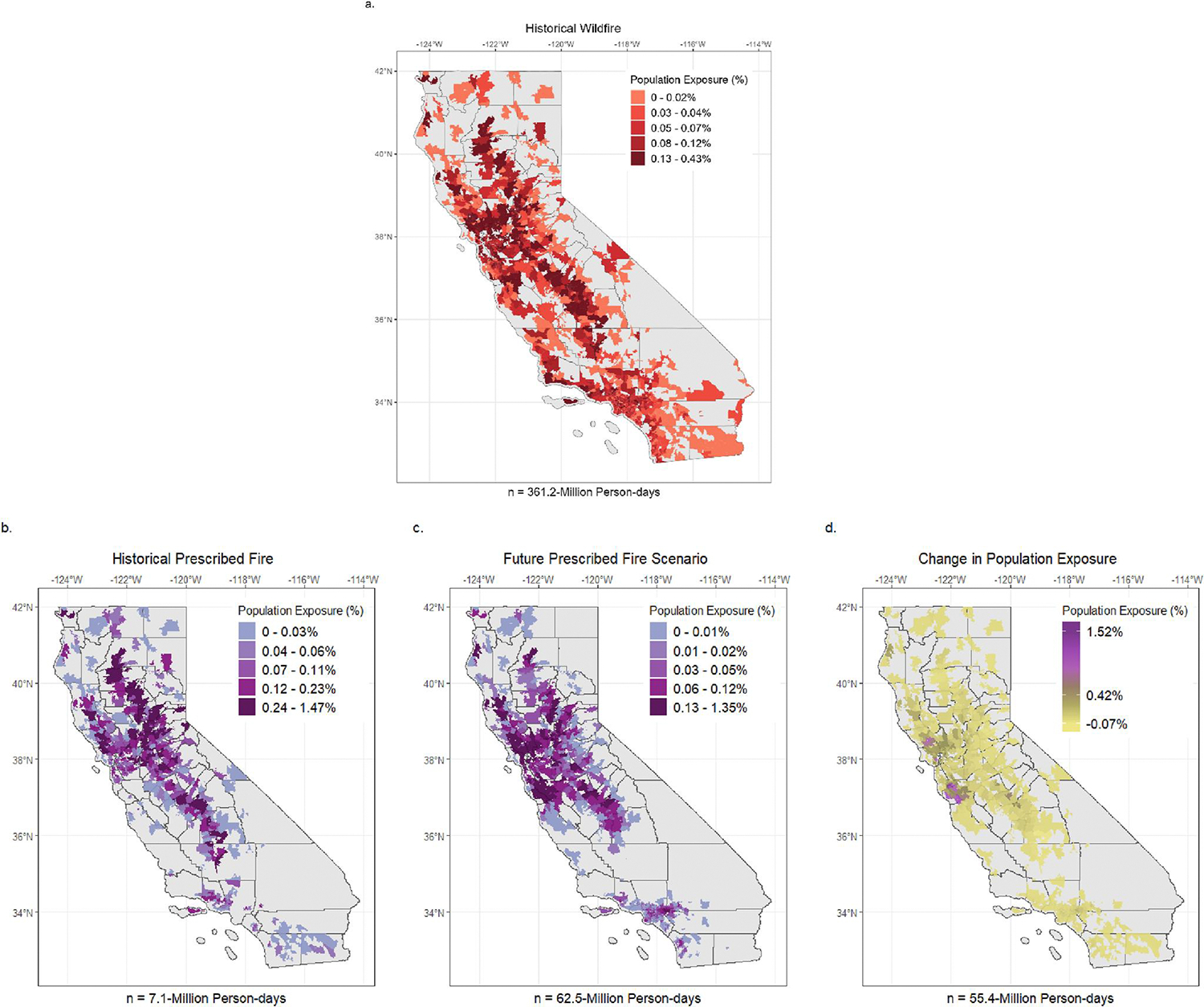
Fraction of Population Exposure on Days with Hybrid Single-Particle Lagrangian Integrated Trajectory (HYSPLIT) Concentrations >5.0 μg/m^3^ during the Historical Period (2008–2016) and Future Prescribed Fire Scenario. Population Exposure is measured as the percent of person-day exposure. Exposures are shown for (a) HYSPLIT-modeled wildfire PM_2.5_ in the Historical Period, (b) HYSPLIT-modeled prescribed fire PM_2.5_ in the Historical Period, and (c) HYSPLIT-simulated prescribed fire PM_2.5_ in the Future Scenario. Panel (d) highlights the change in exposure to prescribed fire between the Historical Period and Future Scenario. Breaks in colors correspond to the quintiles of respective exposure distributions.

The total number of ZIP-days impacted by prescribed fire in the future scenario surpassed the number of ZIP-days impacted by both wildfire and prescribed fire in the historical period. At levels above 5.0 μg/m^3^, exposure (measured in person-days; cumulative number of persons exposed) to future projected prescribed fire smoke was nearly 9x higher than historical prescribed fires and nearly 6x lower than historical wildfires (Table 1). The largest exposure increase was observed in densely populated areas in the northern-central coastal region (Figure 3d). These areas contributed to as much as 1.52% of the total projected increase in person-days of exposure at levels above 5.0 μg/m^3^ (out of 55.4 million additional person-days) in the future scenario compared to historical prescribed fire (Figure 3d). However, exposure was more frequent at lower concentrations, accounting for 99.3% of population exposure to future prescribed fire (Table 1). Note that the 5.0 μg/m^3^ reference value is used solely to describe the magnitude of the difference between higher and lower exposures and is not used to suggest a threshold of effect.

### 3.3. Daily Burden Rates Attributed to Wildfire- and Prescribed Fire-Related PM_2.5_

**Figure 4.**
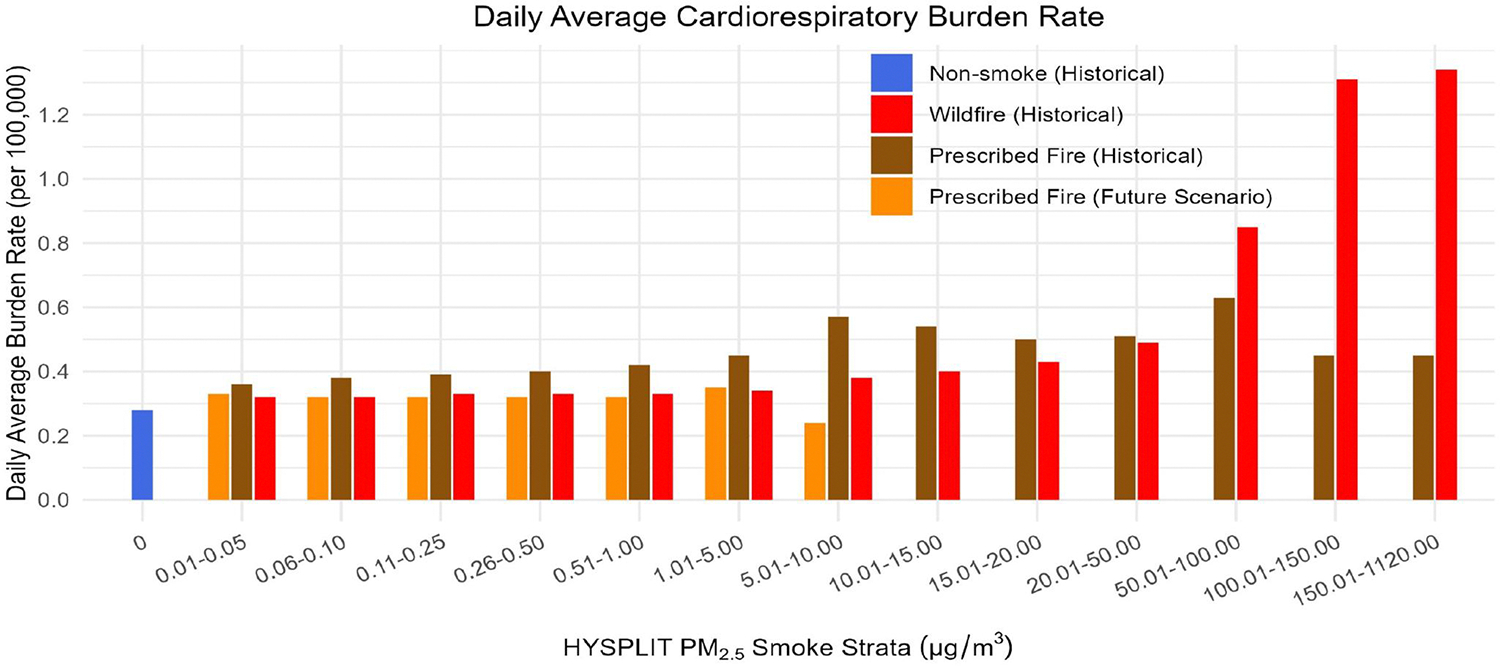
Average Daily PM_2.5_-attributed Burden Rates for Cardiorespiratory Emergency Department Visits by Hybrid Single-Particle Lagrangian Integrated Trajectory-modeled PM_2.5_ Smoke Strata (μg/m^3^). Burden rates are expressed per 100,000 persons. Smoke strata-specific burden rates are shown for no smoke (blue), Wildfire (red) and Prescribed Fire (brown) in the Historical Period, and Prescribed Fire in the Future Scenario (orange).

On days unaffected by smoke (non-smoke days) during the historical period, the daily average cardiorespiratory burden rate was 0.28 per 100K. On smoke-affected days, daily burden rates for lower concentrations of prescribed fire were slightly higher than those observed for wildfires, due to the greater contribution of ambient PM_2.5_ in the specific locations and times of the year where prescribed fires occurred. At the highest smoke concentrations, burden rates from wildfires reflected more significant contributions to ambient PM_2.5_ and exceeded those from prescribed fire. This difference can be attributed to the limited occurrence of high exposure days from prescribed fires, lower incidence rates among the exposed populations, or potential discrepancies between aPM_2.5_ and HYSPLIT Rx-PM_2.5_ estimations. On wildfire or prescribed fire smoke days when concentrations were below 5.0 μg/m^3^, the daily average cardiorespiratory burden rates were between 0.32 and 0.45 per 100K, and up to 1.34 per 100K on wildfire smoke days above 5.0 μg/m^3^ (Figure 4).

In the future scenario, on any given day and in any location affected by smoke, fRx-aPM_2.5_-attributed burden rates were comparable to those in the historical period. Specifically, the average daily fRx-aPM_2.5_ burden rate was approximately 0.32 per 100K at lower levels of exposure (<5.0 μg/m^3^) (Figure 4). Notably, days with higher concentrations of prescribed fire smoke (>10.0 μg/m^3^) had a minimal impact on the statewide average daily burden rates in these stratum (burden rates were lower than two significant figures).

### 3.4. Cardiorespiratory Burden Attributable to Total Ambient PM_2.5_ on Wildfire, Prescribed Fire, and Non-Smoke Impacted Days

**Figure 5.**
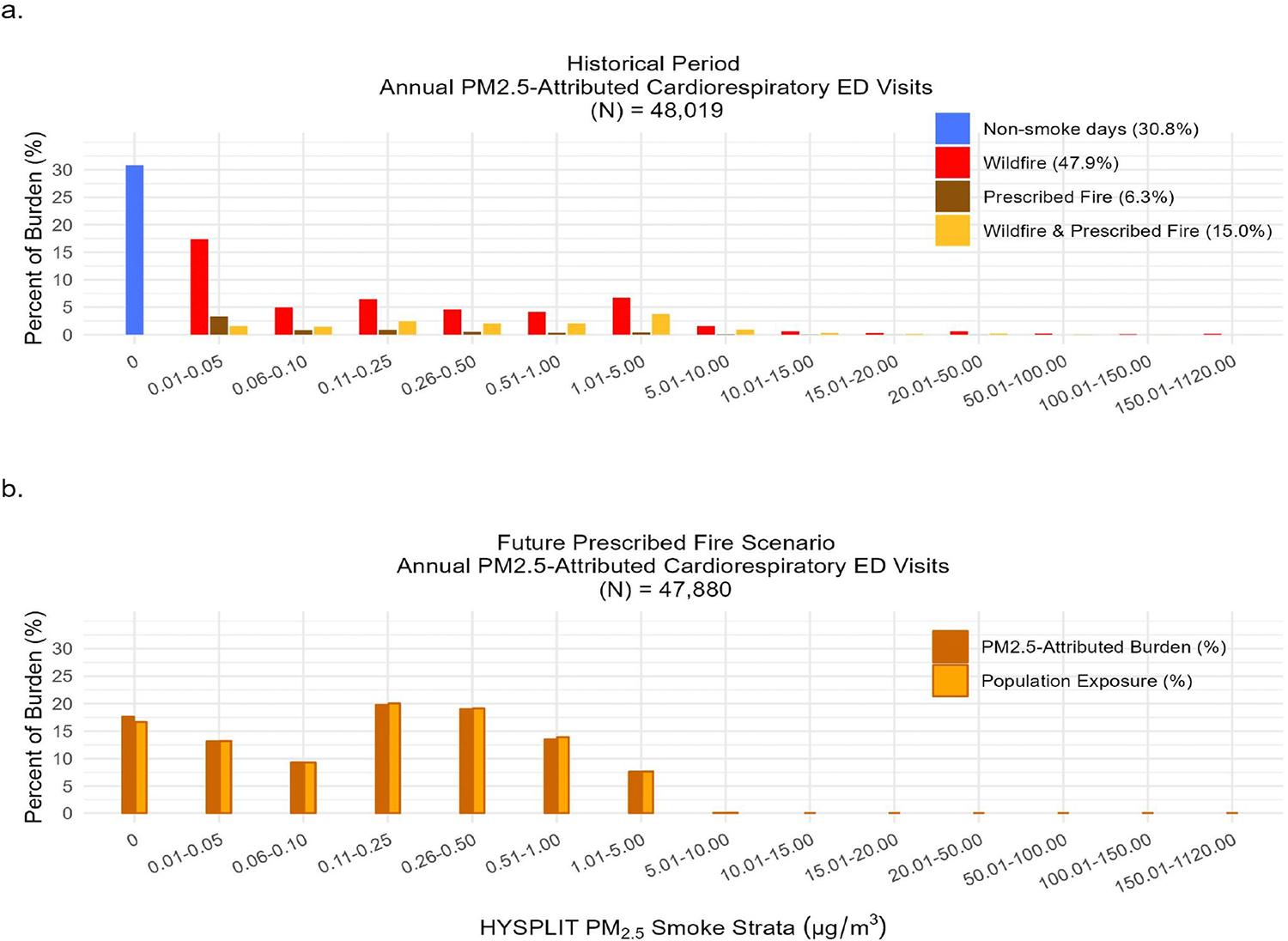
Fraction of PM_2.5_-attributed Cardiorespiratory Emergency Department (ED) Visits by Hybrid Single-Particle Lagrangian Integrated Trajectory (HYSPLIT)-modeled PM_2.5_ Smoke Strata (μg/m^3^). The percent of ED visits is shown for (a) the Historical Period (2008–2016) and (b) the Future Prescribed Fire Scenario (average over 8-cycles) in California. The fraction of population exposure, measured as the percent of person-day exposure by HYSPLIT-modeled PM_2.5_ smoke strata (μg/m^3^), is also shown in (b).

In the historical period, the aPM_2.5_-attributed health burden reflected variations in both exposure and baseline morbidity across different geographic regions. The wildfire smoke-impacted days had the largest attributable ED visit burden (47.9% of 48K visits), followed by non-smoke-impacted days (30.8%), days impacted by both wildfire and prescribed fire smoke (15.0%), and prescribed fire smoke-impacted days (6.3%) (Figure 5a). On wildfire-impacted days, the majority of the aPM_2.5_-attributed burden was associated with lower concentrations of WF-PM_2.5_ (<5.0 μg/m^3^), reflecting the greater frequency of days and the larger affected population at these levels (Figure 5a).

**Figure 6.**
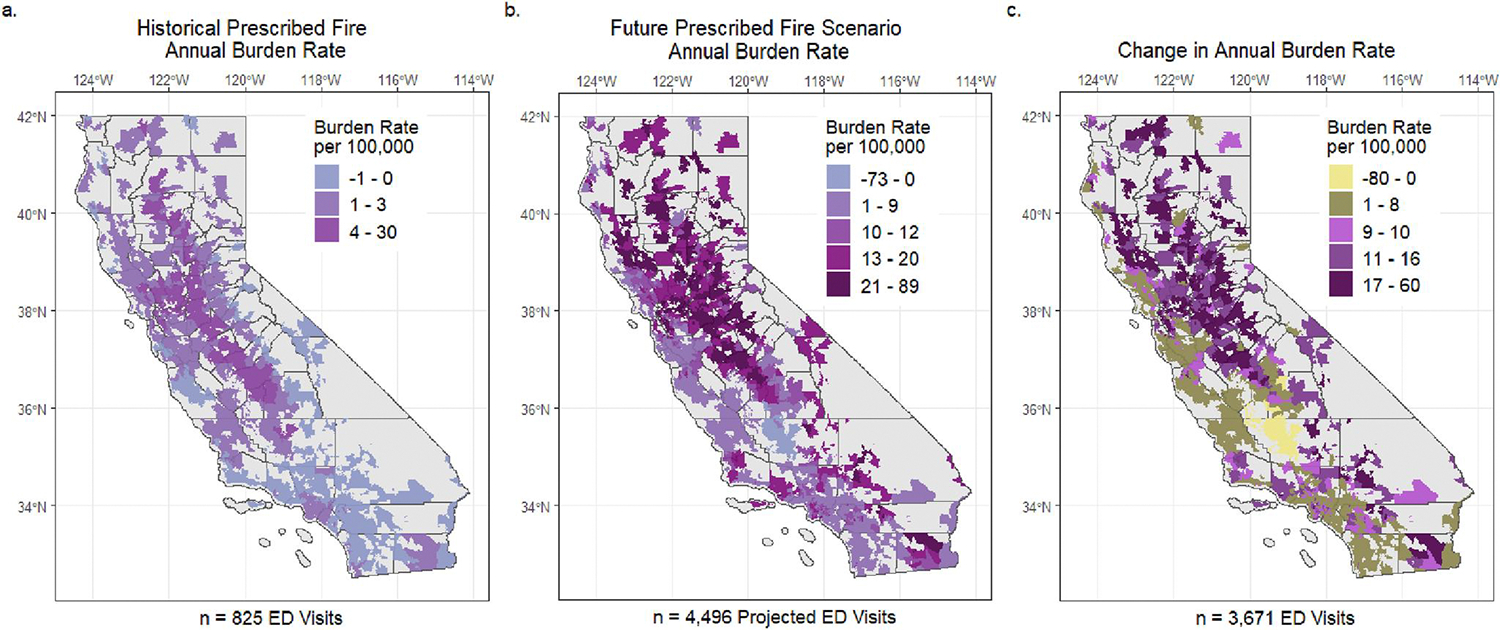
Annual Prescribed Fire Smoke-attributed Cardiorespiratory Burden Rates per 100,000 persons in the Historical Period (2008–2016) and Future Prescribed Fire Scenario. Cardiorespiratory burden rates, measured cumulatively over all days and strata, are shown for (a) Hybrid Single-Particle Lagrangian Integrated Trajectory (HYSPLIT)-modeled Rx-PM_2.5_ exposure in the Historical Period (b) HYSPLIT-simulated Rx-PM_2.5_ exposure in the Future Prescribed Fire Scenario. Panel (c) highlights the change in annual prescribed fire-related burden rates between the Historical Period and Future Scenario. Breaks in colors correspond to the twentieth, 80th, and 100th percentiles in (a) and quintiles (b, c) of respective burden rate distributions.

In the future scenario, we estimated approximately 40K annual aPM_2.5_-attributed ED visits on prescribed fire-impacted days. Nearly all (99.7%) of the aPM_2.5_-attributed burden occurred at concentrations below 5.0 μg/m^3^ (Figure 5b). Cumulatively, over a year, burden rates on prescribed fire smoke days increased above historical levels due to the cumulative impact of more days with lower concentration exposure in densely populated areas. Geographically, we estimated cumulative annual cardiorespiratory burden rates attributed to prescribed fire smoke ranging between 0 and 30 per 100K in the historical period, and between 0 and 89 per 100K in the future scenario (Figures 6a and 6b). We observed the largest increase in cardiorespiratory burden rates in the northern, central, and southern regions of California (Figure 6c). The areas with the most substantial increase in annual burden rates not only experienced more frequent smoke days in the future scenario but were also associated with high daily average levels of ambient PM_2.5_ and a substantial aPM_2.5-_attributed health burden in the historical period. These areas were primarily rural or smaller populations, located in the Central Valley and Northern California.

## 4. Discussion

In this study, we examined past and future fire-smoke exposure scenarios to estimate the anticipated change in PM_2.5_-related health burden from the projected increase in prescribed burning in population-dense, high priority wildfire risk areas of California. The modeling choices represented an aggressive management scenario that achieves the statewide target for prescribed fire and reflects larger impacts in areas with high housing density. Health impacts attributed to prescribed fire smoke were characterized as the difference between excess cardiorespiratory emergency department visits due to ambient all-source PM_2.5_ exposure and the estimated burden under no smoke conditions on the same days and locations. The health burden metric accounted for spatial and temporal variations in the exposure distribution, the frequency and intensity of exposure, in addition to baseline health risks.

### 4.1. Comparing Historical and Future Prescribed Fire Exposures and Burden

For the future prescribed fire scenario, we modeled the projected increase in the number of days impacted by prescribed fire smoke in high-priority areas identified by CAL FIRE as high risk for wildfires. These areas are also characterized as more population-dense than the locations of historical burn activity. The annual exposure to future projected prescribed fire smoke (measured in number of person-days) was approximately 15x more than that of past prescribed fires. At levels above 5.0 μg/m^3^, the exposure was nearly 9x more than that of historical prescribed fires and nearly 6x less than that of historical wildfires (Figures 4a–4c).

In the future scenario, the aPM_2.5_-health burden attributed to prescribed fire smoke increased compared to past prescribed fires, with nearly all the burden attributed to lower levels of exposure. In assessing the change between the historical period and the future prescribed fire scenario, the future projected aPM_2.5_-attributed daily burden rates were similar to, but slightly lower than, those in the historical period. Annually, the increased frequency of exposure days, even at lower concentrations, particularly in densely populated regions, led to a rise in the health burden attributed to prescribed fire smoke. The largest increase in cardiorespiratory burden rates was projected in the broader Central and Northern California regions, encompassing areas that experience high ambient PM_2.5_ concentrations.

### 4.2. Commentary on Current Literature

To our knowledge, this is the first study to characterize the anticipated health burden associated with projected prescribed fire smoke impacts under a future fire management scenario in California. Population-level health impacts of prescribed fire remain largely unknown, as the few studies that have characterized health impacts associated with short-term PM_2.5_ exposure from prescribed burning have done so over shorter time periods with limited spatial coverage (Afrin & Garcia-Menendez, 2021; Huang et al., 2019). Consistent with the findings in this study, Huang et al. (2019) reported that higher estimated health impacts on asthma ED visits were associated with prescribed fire smoke in highly populated areas, despite prescribed fire having a minimal impact on ambient levels of PM_2.5_. Afrin and Garcia-Menendez (2021) similarly noted that areas with higher incidence rates were due to population size and baseline incidence rates.

Methodological approaches in dispersion modeling and exposure assessment differ in the present study from those used in previous studies. Here, we utilized a comprehensively designed dispersion model with larger spatiotemporal coverage to assess prescribed fire smoke impacts on air quality. Therefore, the estimates may have captured greater variability in PM_2.5_ concentrations, plume dispersion, and transport, and may reflect region-specific differences in vegetation type and emissions factors for PM_2.5_, in addition to other dynamics such as prescribed burn regimens and meteorological characteristics, which influence fire behavior, smoke production, and transport (Altshuler et al., 2020; Williamson et al., 2016).

An assessment by US EPA (2021) comparing prescribed fire and wildfire scenarios found that differences in meteorological conditions, the magnitude and duration of fires, and proximity to surrounding populations contributed to variations in smoke exposure and associated health impacts. The assessment reported that health impacts, including estimated respiratory and cardiovascular-related ED visits and hospital admissions, were dominated by wildfire smoke. In addition, the study estimated the potential reduction in health impacts that could be achieved through various smoke-mitigating interventions, reporting that across all scenarios, staying inside, using home HVAC systems, and evacuating an affected area contributed to the largest estimated likelihood of exposure reductions and corresponding smoke PM_2.5_-attributed health events if such actions were employed. Given that prescribed fires are planned, individuals, communities, and fire management personnel may have more opportunity to strategically prepare and mitigate exposure.

### 4.3. Commentary on Toxicity and Health Effects of Wildfire-Sourced Particulate Matter

In this study, we measured the health burden attributed to total ambient PM_2.5_, stratified by ranges of smoke concentrations. In doing so, we made a simplified assumption that the underlying biological mechanisms associated with PM_2.5_ exposure and cardiorespiratory health effects are similar between particles emitted from different sources. The authors did not believe that differential toxicity can be inferred based on the available data at the time the study was conducted. Whereas many analyses of air pollution source impacts rely on existing concentration-response functions extracted from studies conducted at separate times and locations, this study applied concentration-response functions and incidence rates based on the same period and population under investigation.

The high contribution to ambient PM_2.5_ concentrations from wildfire smoke underlies the difficulty in discerning the differential toxicity between multi-sourced and biomass-sourced ambient particles in population studies. Epidemiological studies have primarily examined the health effect of wildfire smoke by analytically separating anthropogenic PM_2.5_ from smoke emissions using chemical transport models or by subtracting out historically observed averages, while others have separated exposure to PM_2.5_ on days with smoke versus days without smoke (DeFlorio-Barker et al., 2019; Gan et al., 2017; Liu et al., 2017). We adapted the latter approach, stratifying the distribution of all-source PM_2.5_ exposure and associated health impacts by concentrations of smoke PM_2.5_ estimated by the HYSPLIT model. We then characterized the portion of the total PM_2.5_ burden attributed to prescribed fire smoke. We reason that populations are exposed to entire PM mixtures in ambient settings and that both smoke-derived PM_2.5_ and ambient multi-source PM_2.5_ contribute to the total potency of the mixture.

Particle pollution health studies support the determination of US EPA’s Integrated Scientific Assessment for Particulate Matter that for both short- (days to weeks) and long-term (months to years) PM_2.5_ exposure, there is a “causal relationship” for cardiovascular effects and mortality and “likely to be a causal relationship” for respiratory effects (EPA, 2020). Previous wildfire health studies have found that PM_2.5_ from wildfire smoke is more strongly associated with respiratory effects compared to PM_2.5_ from other sources (Aguilera et al., 2021; DeFlorio-Barker et al., 2019; Reid et al., 2016) and recent evidence suggests positive associations between wildfire smoke and cardiovascular effects (Dennekamp et al., 2015; Haikerwal et al., 2015; Jones et al., 2020; Wettstein et al., 2018). Although wildfire smoke epidemiology is a rapidly growing area of research, the scientific knowledge about the health risks and biological mechanisms through which particle pollution exposure exacerbates health remain rooted in decades of ambient air pollution research largely based on total ambient PM_2.5_ emissions.

Several factors should be considered when generalizing health effects from ambient air pollution to effects from wildland fires. First, during wildfire events, populations are exposed to a complex mixture of particles and gaseous chemicals at high concentrations due to biomass combustion. This mixture differs from the multiple sources of combustion and particle mixtures present in the ambient setting, potentially resulting in different effects (e.g., synergistic or additive effects). Second, population exposure patterns and behavioral responses may also differ during wildfire events compared to typical ambient air pollution exposure from other sources. Moreover, wildfires situated in the wildland urban interface (WUI) may burn built infrastructure in addition to vegetation, which likely shifts the PM_2.5_ mixture from those created solely through the combustion of natural biomass fuels to emissions from the burning of often more toxic materials (EPA, 2021). Taken together, these factors can contribute to differential toxicity between smoke and ambient pollution, but also between wildfire and prescribed burning.

Toxicological studies in particular provide the strongest evidence of the differential toxicity of fire-derived PM_2.5_ based on the types of fuel source and burn conditions of a particular area (Kim et al., 2018). However, when evaluating the population health impact of smoke exposure, the composition of particle mixtures may be of secondary public health concern relative to the overall toxicity and concentrations of a smoke mixture.

### 4.4. Commentary on Limitations

Several limitations of this study should also be noted. Past records of prescribed fire are considered incomplete. However, our data set integrated multiple data sources and we believe is as robust as possible for historical data. If the lack of complete records led to an underestimation of past prescribed burning, then the magnitude of the relative future burden increase may be lower. Fortunately, enhancing the data tracking systems for prescribed fire in California is part of the current effort to expand the use of prescribed fire; therefore, future studies will have access to much more complete data (California Wildfire & Forest Resilience Task Force, 2022; Forest Management Task Force, 2021). This study focused on fire management practice, and as such, does not address PM_2.5_ impacts due to agricultural burning in California. However, emissions from this source would be captured using ambient all-source PM_2.5_ measures and thus are incorporated into our health burden estimates.

The ensemble model used to estimate the daily average total ambient PM_2.5_ (aPM_2.5_) data utilized in this study may have underestimated exposure concentrations on days with very high levels of air pollution. Considine et al. (2023) recently reported regional differences in model performance in the Western US and noted that variability of PM_2.5_ concentrations was underestimated in areas impacted by high concentrations of wildfire smoke. Therefore, burden rates associated with extreme smoke events (very high concentrations of wildfire or prescribed fire smoke exposure) may be higher than those estimated.

The use of administrative health data to identify cardiorespiratory-related ED events may be subject to outcome misclassification. ICD diagnosis codes associated with patient-level emergency department visits are used for billing purposes and may not capture or define disease as accurately as medical records (O’Malley et al., 2005). Second, the use of residential ZIP codes to assign modeled PM_2.5_ concentration estimates may be a source of exposure misclassification. However, given the lack of availability of health data at a finer spatial scale, residential ZIP codes represent a proxy for personal exposure in studies of air pollution at ecological scale. To mitigate the impact of exposure misclassification, daily average concentrations were population-weighted by ZIP code. Additionally, population data used in this study are based on 2010 census estimates; therefore, future projected health burden estimates did not account for future population trends in California, which may underestimate the health burden due to population increases over time. However, the modeling choice to focus on more populated areas would increase projected burden estimates.

As in all air pollution epidemiologic studies, the possibility of unmeasured confounding bias cannot be ruled out. Data on pre-existing health conditions or individual-level measures of socioeconomic status were not available in our analysis, which may be important confounders affecting PM_2.5_-attributed health impacts. We did not account for individual- or community-level health-related behaviors such as smoking, diet, and exercise to examine how adjustment for these risk factors may affect risk estimates, which may not have captured the extent of variability in health behaviors, geographically. On the other hand, it is possible that the intensity of exposure concentrations coupled with public perceptions of wildfires compared to prescribed fire may lead to differences in the likelihood of undertaking smoke-mitigating actions or health-protective behaviors that reduce the impact of smoke exposure. Assessments in these directions will greatly benefit future research.

In the future scenario, prescribed fires were deliberately simulated within CAL FIRE-designated high-priority areas for wildfire risk mitigation. Therefore, focusing the model on these densely populated WUI areas is anticipated to result in a larger health burden compared to a model centered on remote, sparsely populated, high wildfire-risk areas where prescribed fires were historically more prevalent. Moreover, the target scenario did not account for the maintenance or recurrent burns required to sustain the efficacy of prescribed fires in mitigating wildfire risk. This is because the primary focus of the study was to assess the impacts of increased burning at the target scale, rather than evaluating fuel treatment strategies. Additionally, the fire size and fuels selected for the hypothetical future prescribed burns could significantly differ from present-day or future prescribed burning patterns, potentially making them unrealistic. The reported results should be interpreted within the context of a specific fire management scenario, based on historical patterns of prescribed fire and meteorological conditions. Future climate and wildfire activity are uncertain, which is likely to alter land and fire management practices. Further analyses exploring the impact of burn sizes will benefit future research.

Due to the unpredictable nature of future wildfire events, this study did not attempt to predict potential reductions in future wildfire emissions and the associated health benefits resulting from the increased use of prescribed fire. Nevertheless, the study enables a comparison between the anticipated health burden of increased prescribed fire at a projected scale in high-risk areas, and the impacts of wildfires and prescribed fires as observed in the recent historical period. Furthermore, it’s worth noting that this analysis spans wildfire events up until 2016. More recent wildfires events in California (2017–2021) likely exhibited a higher occurrence of extreme smoke events (Higuera & Abatzoglou, 2021), leading to more days and prolonged periods of high PM_2.5_ exposure affecting large populations in California. Therefore, the omission of more recent wildfire seasons in this study might not completely capture the magnitude of the difference in potential impacts between wildfire and prescribed fire.

Estimating the atmospheric transport of smoke emissions is complex; variation in the impact and dispersion of emissions are influenced by many factors including meteorological conditions (wind speed and direction), fuel loads, timing, and seasonality (Liu & Peng, 2019). The HYSPLIT dispersion models used in this study do not consider chemical processes. Therefore, secondary formation of aerosols from smoke emissions are not accounted for in the model output. This would limit the correlation of modeled smoke PM_2.5_ concentrations with measured total PM_2.5_ concentrations. As described in the methods, future projected prescribed fire smoke emissions data were developed from dispersion model simulations that used 1 year of meteorological data for computational efficiency, with the year 2014 selected because it was most representative of favorable meteorological conditions for prescribed burning over the historical period. Lastly, this analysis only considered single day prescribed burn events, which reflects common practice, although in the future, burn regimes may be multi-day events.

## 5. Conclusion

This study was undertaken to gain an understanding of the potential public health implications resulting from fire management practices that are increasing the use of prescribed fire in California.

We established a framework that integrates principles of epidemiology and risk assessment with simulations of future prescribed fire scenarios, enabling the quantification and comparison of health impacts. The health burden metric employed in this analysis accounted for spatial and temporal variations in exposure, the frequency and intensity of exposure, as well as baseline health risks. The California-based model, simulating the hypothetical increase of prescribed fire in high-priority wildfire risk areas, indicated that a larger population will experience smoke. The increase in exposure days led to an overall increase in the health burden in the future scenario compared to the past. Notably, the excess future health burden was due to the accumulation of more frequent lower-concentration exposure days and high population density in high-priority wildfire risk areas. The analytical approach can be extended in future studies to identify populations at highest risk, with a particular focus on factors such as age, race/ethnicity, socioeconomic status, and other indicators of health equity. Characterizing and` mitigating the potential impact of prescribed fire smoke will help realize the benefits of prescribed fire and optimize its effective implementation for public health protection as well as safety from wildfires.

## Supplementary Material

Supplement1

## Figures and Tables

**Tablel:** Annual Number of ZIP‐Days Impacted by Smoke and Annual Number of Person‐Days of Exposure in the Historical Period (2008–2016) and Future Prescribed Fire Scenario in California

	Historical						Future	
	Total ambient PM_2.5_ (aPM)		Prescribed fire (Rx-PM)		Wildfire (WF-PM)		Prescribed fire (fRx-PM)	
PM_2.5_ (μg/m^3^)	All ZIP-Days	Person-days in Millions (%)	Rx ZIP-Days	Person-days in Millions (%)	WF ZIP-Days	Person-days in Millions (%)	fRx ZIP-Days	Person-days in Millions (%)

0.01–0.05	9	0.1 (0.001)	14,792	398.8 (56.7)	78,439	2,324.5 (37.2)	83,875	2,387.9 (22.8)
0.06–0.10	17	0.3 (0.002)	3,300	87.8 (12.5)	22,709	663.5 (10.6)	47,099	1,341.2 (12.8)
0.11–0.25	88	0.9 (0.007)	3,548	91.9 (13.1)	29,831	863.6 (13.8)	83,584	2,381.4 (22.7)
0.26–0.50	520	5.0 (0.04)	2,015	49.4 (7.0)	21,124	607.3 (9.7)	65,366	1,875.8 (17.9)
0.51–1.00	3,000	30.4 (0.2)	1,440	33.3 (4.7)	19,466	553.9 (8.9)	48,972	1,400.6 (13.4)
1.01–5.00	119,525	2,353.9 (17.6)	1,610	34.8 (5.0)	31,642	866.9 (13.9)	37,621	1,026.2 (9.8)
5.01–10.00	194,353	5,691.1 (42.5)	236	4.4 (0.6)	7,135	185.2 (3.0)	1,975	47.2 (0.5)
10.01–15.00	100,017	3,396.7 (25.4)	66	1.3 (0.2)	2,723	67.9 (1.1)	387	9.3 (0.1)
15.01–20.00	30,950	1070.7 (8.0)	24	0.4 (0.1)	1,364	33.0 (0.5)	132	3.3 (0.03)
20.01–50.00	23,849	802.4 (6.0)	36	0.5 (0.1)	2,341	53.6 (0.9)	114	2.7 (0.03)
50.01–1120.00	1,376	41.0 (0.3)	24	0.6 (0.1)	1,159	21.5 (0.3)	5	0.1 (0.001)
Person-days in Billions		13.4 (100)		0.7 (100)		6.2 (100)		10.5 (100)
Total Number of ZIP- Days	473,704		27,091		217,933		369,130	

*Note.* The table summarizes the annual number of days across ZIP codes impacted by smoke (smoke days) and average annual number of person‐days of exposure to all‐source fine particulate matter (total ambient PM_2.5_ [aPM_2.5_]), Hybrid Single‐Particle Lagrangian Integrated Trajectory (HYSPLIT)‐modeled prescribed fire (Rx‐PM_2.5_) and wildfire (WF‐PM_2.5_) smoke PM_2.5_, between 2008 and 2016, and HYSPLIT‐simulated future prescribed fire smoke PM_2.5_ (fRx‐PM_2.5_) concentrations (μg/m^3^) averaged over 8‐annual‐cycles in California ZIP codes.

## Data Availability

The data sets presented in this study can be found in online repositories. Daily average ambient PM_2.5_ concentrations (μg/m^3^) were obtained from Di et al. (2019). The wildfire and prescribed fire emissions data used for analysis in this study are available from Rosenberg et al. (2023). The Projected Future Prescribed Fire Scenario data used for analysis in this study are available from Kramer et al. (2023). Statewide emergency department (ED) visit records were obtained from the California Department of Health Care Access and Information (HCAI). HCAI provides confidential patient-level data sets to researchers eligible through the Information Practices Act (CA Civil Code Section 1798 et seq.), which permits nonprofit educational institutions and state agencies to request data for research purposes and for performing legally mandated activities. https://hcai.ca.gov/data-and-reports/research-data-request-information/. Please note, raw health data is not provided due to confidentiality of personal health information used for research. Aggregated estimates of PM_2.5_-attributed ED visit counts and rates per 100,000 are provided at a restricted resolution. R Statistical Software (R version 4.2.3; R Core Team, 2021) was used to execute and report on all analyses in this paper. All scripts used to prepare and analyze the data are provided in Rosenberg (2023).

## Key Points:

- A California-based model of future prescribed burning in high-priority wildfire risk areas suggested more people will experience smoke
- An increased number of exposure days in the future scenario led to an overall increase in the future health burden
- The excess future health burden was due to the cumulative impact of lower exposure days and high population density in high-priority areas
