# Supplementary material for: Health Impacts of Future Prescribed Fire Smoke: Considerations From an Exposure Scenario in California
Source: Earths Future. Author manuscript; Available in PMC 2025 Jun 21. (PMC12181949; doi:10.1029/2023EF003778)
Supplement: Supplement1 [file NIHMS2073169-supplement-Supplement1.pdf]

Earth's Future

Supporting Information for

Health Impacts of Future Prescribed Fire Smoke: Considerations from an Exposure Scenario in California

Andrew Rosenberg,[1*] Sumi Hoshiko,[1*] Joseph R. Buckman,[1,2] Kirstin R. Yeomans,[1,2*] Thomas Hayashi,[1,3] Samantha J. Kramer,[4*] ShihMing Huang,[4*] Nancy H.F. French,[5*] Ana G. Rappold[6†*]

[1]Environmental Health Investigations Branch, California Department of Public Health, Richmond, California, USA
[2]California Epidemiologic Investigation Service Fellowship Program (Cal-EIS), Chronic Disease Control Branch, Center for Chronic Disease Prevention and Health Promotion, California Department of Public Health, Sacramento, California, USA
[3]Sequoia Foundation, La Jolla, California, USA
[4]Sonoma Technology, Inc., Petaluma, California, USA
[5]Michigan Tech Research Institute, Michigan Technological University, Ann Arbor, Michigan, USA
[6]Center for Public Health and Environmental Assessment, United States Environmental Protection Agency, Durham, North Carolina, USA.

Corresponding author: Ana G. Rappold, PhD (Rappold.ana@epa.gov)

[†]Center for Public Health and Environmental Assessment
Office of Research and Development
U.S. Environmental Protection Agency
Research Triangle Park, NC, USA

Contents of this file

Text S1
Tables S1 to S4
Figures S1 to S3

Introduction

The supporting information includes details about the evaluation of the HYSPLIT model $PM_{2.5}$ concentration estimates. Summaries of exposure distributions of total ambient $PM_{2.5}$ ($aPM_{2.5}$) data (2008-2016), HYSPLIT-modeled wildfire PM2.5 (WF-$PM_{2.5}$) (Historical; 2008-2016), HYSPLIT-modeled prescribed fire $PM_{2.5}$ (Rx-$PM_{2.5}$) (Historical; 2008-2016), and HYSPLIT-simulated prescribed fire $PM_{2.5}$ in the Future Prescribed Fire Scenario (fRx-$PM_{2.5}$) (8-annual cycle/years). Exposure is summarized by percentile, HYSPLIT PM2.5 Smoke Strata, and annually for each fire type. In addition, a figure comparing the annual mean of prescribed fire PM2.5 on smoke-impacted days, along with a map depicting the distribution of smoke days in both the Historical Period and Future Prescribed Fire Scenario, is provided. Lastly, a reference map CAL FIRE designated Community Wildfire Threat Priority Landscapes in California is included.

Text S1. Evaluation of BlueSky-HYSPLIT estimated $PM_{2.5}$ concentrations.

Evaluation of BlueSky-HYSPLIT $PM_{2.5}$ concentration estimates was conducted by examining the correlation between predicted concentrations and measurements at routine $PM_{2.5}$ monitoring stations (US EPA's Air Quality System and Interagency Monitoring of Protected Visual Environments [IMPROVE) sites), with and without using satellite smoke plume detection (NOAA's Hazard Mapping System [HMS] smoke product) as a filter. Correlation was chosen because the modeled concentrations included smoke contributions only while measurements contain total $PM_{2.5}$ from all sources. We found positive moderate and variable correlations between modeled baseline $PM_{2.5}$ concentration with AQS data (r=0.31-0.37) and IMPROVE data (r=0.18-0.68) respectively. Modeled prescribed fire $PM_{2.5}$ correlated with measurements data (r=0.37-0.68) better than wildfire and prescribed fire-combined $PM_{2.5}$ did (r=0.2-0.32), and even better than wildfire $PM_{2.5}$ alone did (r=0.18-0.31). These relationships are reasonable considering that modeled fire $PM_{2.5}$ and measured total $PM_{2.5}$ are related but not equivalent; these are furthermore comparable to Yao et al. (2013) (r=0.31-0.4), Yuchi et al. (2016) (r=0.58), and Michael et al. (2023) (ρ=0.18-0.34).

Table S1. Exposure distribution of daily mean all-source fine particulate matter (Total ambient $PM_{2.5}$) on all days, HYSPLIT-modeled historical wildfire derived $PM_{2.5}$ (WF) and prescribed fire (Rx) derived $PM_{2.5}$ (2008-2016), and HYSPLIT-simulated future prescribed fire-derived $PM_{2.5}$ (8-annual-cycles) ($\mu g/m^3$) on days impacted by smoke in California ZIP codes. Total ambient $PM_{2.5}$ is daily ambient $PM_{2.5}$ from all sources, 2008-2016.

	**Historical**			**Future**
Percentile (%)	Total $PM_{2.5}$ (All days) (n=4,263,239)	Wildfire $PM_{2.5}$ (n=1,961,396)	Prescribed Fire $PM_{2.5}$ (n=243,763)	Prescribed Fire $PM_{2.5}$ (n=2,953,029)
0	0.01	0.01	0.01	0.01
20	4.31	0.02	0.01	0.05
30	5.45	0.04	0.02	0.08
40	6.55	0.07	0.03	0.13
50	7.70	0.13	0.04	0.19
60	8.98	0.25	0.07	0.27
70	10.48	0.51	0.13	0.40
80	12.45	1.12	0.26	0.61
90	15.93	3.17	0.69	1.07
95	20.46	6.94	1.54	1.71
97.5	26.05	13.58	3.03	2.60
99	34.64	29.54	6.56	4.22
100	175.26	1119.73	252.79	83.05

*Total ambient $PM_{2.5}$ is daily mean all-source ambient $PM_{2.5}$ per ZIP code in California, 2008-2016 (Di et al., 2019)

Table S2. Cumulative number of days impacted by wildfire and prescribed fire smoke (Historical Period - 2008-2016) and future projected number of days impacted by prescribed fire (Future Scenario – 8-annual-cycles) per stratum of smoke-derived $PM_{2.5}$ concentrations (μg/m^3) in California ZIP codes.

	**Historical**		**Future**
$PM_{2.5}$ (μg/m^3)	Wildfire (%)	Prescribed Fire (%)	Prescribed Fire (%)
0.01-0.05	705,949 (36.0)	133,131 (54.6)	671,001 (22.7)
0.06-0.10	204,385 (10.4)	29,698 (12.2)	376,789 (12.8)
0.11-0.25	268,483 (13.7)	31,930 (13.1)	668,675 (22.6)
0.26-0.50	190,114 (9.7)	18,132 (7.4)	522,927 (17.7)
0.51-1.00	175,190 (8.9)	12,957 (5.3)	391,772 (13.3)
1.01-5.00	284,776 (14.5)	14,492 (5.9)	300,971 (10.2)
5.01-10.00	64,217 (3.3)	2,126 (0.9)	15,797 (0.5)
10.01-15.00	24,508 (1.2)	595 (0.2)	3,093 (0.1)
15.01-20.00	12,275 (0.6)	212 (0.1)	1,056 (0.04)
20.01-50.00	21,065 (1.1)	321 (0.1)	911 (0.03)
50.01-100.00	5,737 (0.3)	100 (0.04)	37 (0.001)
100.01-150.00	1,924 (0.1)	34 (0.01)	0
150.01-253.00	1,578 (0.1)	35 (0.01)	0
253.01-1120.00	1,195 (0.1)	0	0
**Total**	1,961,396	243,763	2,953,029

Table S3. Exposure distribution of total ambient fine particulate matter ($PM_{2.5}$) (μg/m^3) on wildfire and prescribed fire smoke-impacted ZIP code days (smoke-days*) and days not impacted by smoke in California, 2008-2016.

	**Total $PM_{2.5}$**		
Percentile (%)	Non-smoke Days	Wildfire Smoke days	Prescribed Fire Smoke days
0	0.01	0.03	0.02
20	3.36	5.32	3.58
30	4.25	6.49	4.73
40	5.16	7.60	5.89
50	6.13	8.73	7.13
60	7.22	9.95	8.55
70	8.54	11.34	10.44
80	10.31	13.11	13.30
90	13.42	16.17	19.53
95	17.27	20.12	25.66
97.5	21.94	25.44	31.34
99	29.03	34.63	38.72
100	175.26	174.53	141.04

Table S4. Annual mean ambient $PM_{2.5}$, HYSPLIT-modeled wildfire-derived $PM_{2.5}$ (WF-$PM_{2.5}$), and HYSPLIT-modeled prescribed fire-derived $PM_{2.5}$ (Rx-$PM_{2.5}$) (μg/m^3) in California, 2008-2016.

Year	Total ambient $PM_{2.5}$ (μg/m^3)	HYSPLIT WF-$PM_{2.5}$ (μg/m^3)	HYSPLIT Rx-$PM_{2.5}$ (μg/m^3)
2008	11.11	3.80	0.10
2009	10.07	0.84	0.20
2010	8.71	0.36	0.11
2011	9.14	0.71	0.13
2012	8.82	0.49	0.06
2013	9.31	1.21	0.15
2014	8.77	1.14	0.06
2015	8.61	0.98	0.12
2016	7.46	0.54	0.06

Figure S1. Comparison of annual mean prescribed fire-derived $PM_{2.5}$ (Rx-PM) on Rx smoke-impacted days in the historical (2008-2016) and future projected (8-yrs/cycles) prescribed fire (fRx-$PM_{2.5}$) scenario in California.

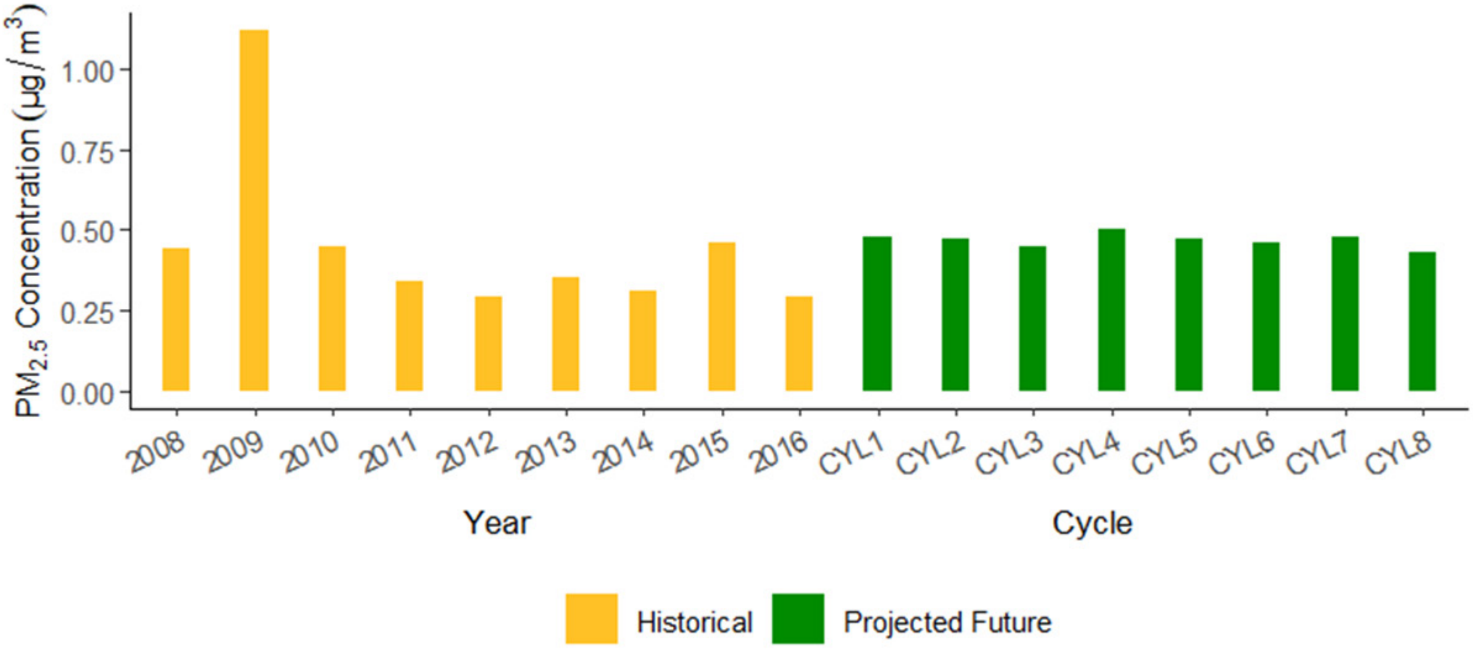

Figure S2. Distribution of Prescribed Fire Smoke Days in the Historical Period (2008-2016) and Future Scenario in California. Breaks in colors correspond to the quintiles of respective distributions. A smoke day is defined as any ZIP-day where Rx-$PM_{2.5} > 0$.

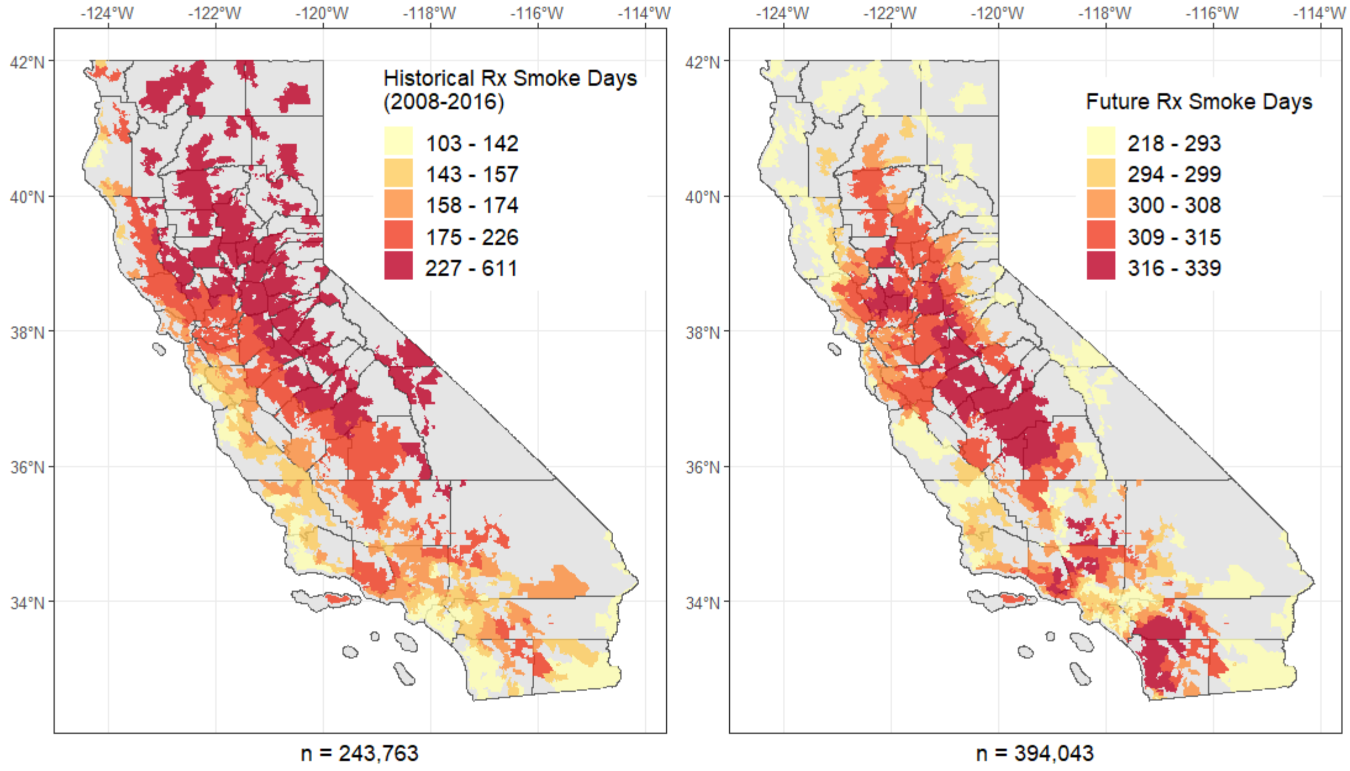

Figure S3. Map of Community Wildfire Threat Priority Landscapes in California. Priority Landscape Classes 4 and 5 are the orange and red shaded areas. The risk to communities is defined by a combination of housing density (housing unit/acre) in the Wildland Urban Interface (WUI) and by threat of Fire Hazard Severity to communities. Higher density areas are ranked higher than less dense areas. Threat is assessed using California Fire Hazard Severity Zones (Moderate to very high severity). The final ranking varies from 1 (least risk) to 5 (greatest risk).

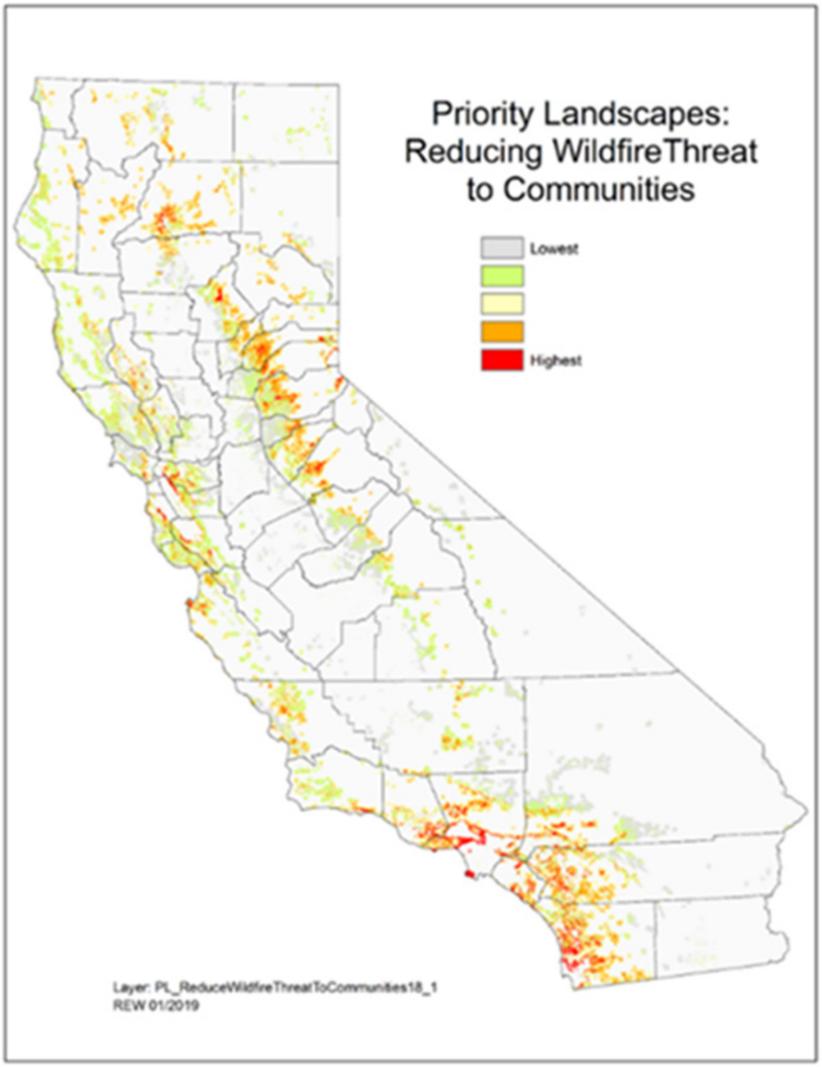

Figure 2. Reducing Wildfire Threat to Communities (2018)
